# Endophytic *Bacillus* spp. as a Prospective Biological Tool for Control of Viral Diseases and Non-vector *Leptinotarsa decemlineata* Say. in *Solanum tuberosum* L.

**DOI:** 10.3389/fmicb.2020.569457

**Published:** 2020-10-15

**Authors:** Antonina Sorokan, Ekaterina Cherepanova, Guzel Burkhanova, Svetlana Veselova, Sergey Rumyantsev, Valentin Alekseev, Ildar Mardanshin, Elena Sarvarova, Ramil Khairullin, Galina Benkovskaya, Igor Maksimov

**Affiliations:** ^1^Laboratory of Biochemistry of Plant Immunity, Institute of Biochemistry and Genetics, Ufa Federal Research Center, Russian Academy of Sciences, Ufa, Russia; ^2^Laboratory of Genomics of Plants, Ufa Federal Research Center, Institute of Biochemistry and Genetics, Russian Academy of Sciences, Ufa, Russia; ^3^Laboratory of Selection and Seed Production of Potato, Bashkir Research Institute of Agriculture, Ufa Federal Research Center, Russian Academy of Sciences, Ufa, Russia; ^4^Laboratory of Physiological Genetics, Institute of Biochemistry and Genetics, Ufa Federal Research Center, Russian Academy of Sciences, Ufa, Russia

**Keywords:** *Solanum tuberosum*, viruses, endophyte, *Bacillus*, RNases

## Abstract

Viral diseases and their damage causing significant loss to economically important crops have increased by several folds during the last decade. All the conventional approaches are not able to eradicate the viral infection. Therefore, there is a need to look for efficient and eco-friendly viral disease-preventive measures. The genomic material of the majority of deleterious viruses of higher plants is RNA. One of the possible measures to control viruses is the use of ribonucleases (RNases), which can cleave RNA in the viral genome. Based on this, we investigated the RNase activity of endophytic *Bacillus* spp., which can enrich in 10^3^–10^5^ colony-forming units per gram of wet mass of aboveground part of potato plants. A high level of RNase activity was observed in the culture medium of *Bacillus thuringiensis* B-6066, *Bacillus* sp. STL-7, *Bacillus* sp. TS2, and *Bacillus subtilis* 26D. *B. thuringiensis* B-5351 had low RNase activity but high ability to colonize internal plant tissues, *Bacillus* sp. STL-7 with high RNase activity have relatively low number of cells in internal tissues of plants. *B. thuringiensis* B-6066, *B. subtilis* 26D, and *Bacillus* sp. TS stimulate RNase activity in potato plants for a long time after application. Strains with high ability to colonize internal plant tissues combined with high RNase activity reduced severity of viral diseases symptoms on plants and reduced the incidence of potato viruses M, S, and Y. It is worth noting that *Bacillus* spp. under investigation reduced the number of *Leptinotarsa decemlineata* Say. egg clusters and larvae on treated plants and showed antifeedant activity. This results in increase of potato productivity mainly in the fraction of major tubers. *B. subtilis* 26D and *Bacillus* sp. TS2 combining endophytic lifestyle, RNase, and antifeedant activity may become the basis for the development of biocontrol agents for plant protection.

## Introduction

Viruses cause epiphytoties among all agricultural crops worldwide. This threatens food security and stability of crop yields in a number of regions. The development of efficient and durable resistance able to withstand viral attacks represents a major challenge for agrobiology. For instance, cultivated plants are affected by at least 450 different viruses (Soosaar et al., 2005), more than 40 of them infect potato plants (*Solanum tuberosum* L.), significantly reducing their productivity and deteriorating the quality of tubers (Makarova et al., 2017), which is known as the cultivar degeneration. The most common and important viruses of potato are potato leaf roll virus (PLRV), potato virus Y (PVY), potato virus A (PVA), potato virus X (PVX), potato virus S (PVS), and potato virus M (PVM). Currently, viruses cannot be controlled with chemical pesticides, since known antiviral compounds such as teratogenic ribavirin (1,β-D-ribofuranosyl-l,2,4-triazole-3-carboxamide) are hazardous to people’s health (De Fazio et al., 1980). Considering that viruses have more than one species of plant host and that several unrelated viruses can infect plants simultaneously, it is important that more than one virus can be found within the same plant and can damage crops to a greater degree (Moreno and López-Moya, 2020). Thus, PVY is the most important viral pathogen in the world. PVY caused major economic damage to potato production worldwide (Alyokhin et al., 2008). In contrast to vector-borne viruses, PVY can be transmitted both non-persistently by aphids and mechanically through contact with infected plants. Thus, insecticides are an inadequate method of PVY control (Hussain et al., 2016). At the moment, PVY rapidly evolving and an increasing number of strains in PVY-complex evade many certification practices (based on PCR or immunoassay analysis) and defense measures. Seed certification programs often cannot be effective at managing PVY below economic thresholds as a consequence of its variability (Gray and Power, 2018). Yield losses caused by PVS and PVM are usually less than 10–20%, but co-infections of PVY and PVS and/or PVM amplify PVY caused losses (Pruss et al., 1997). Unfortunately, the latter is common in potatoes in many regions.

Non-vector herbivores, such as the most harmful pest Colorado potato beetle (CPB; *Leptinotarsa decemlineata* Say.) can mechanically transfer potato viruses. Diverse life cycle, ecological mobility, symbiosis with bacterial species (Sorokan et al., 2019), and high adaptability to a broad spectrum of stressors, including pesticides, allow CPB to spread almost everywhere (Alyokhin et al., 2008). Control of CPB is a difficult task since the pest becomes resistant to pesticides. Increased growth of CPB larvae, which propagated on PVY-infected plants in comparison with uninfected plants, was reported previously (Kersch-Becker and Thaler, 2014; Petek et al., 2014). Non-vectored tobacco mosaic virus (TMV) improved the survival of CPB larvae and adults, possibly, due to the increased nitrogen content in TMV-infected plants (Hare and Dodds, 1987). CPB female adults tended to feed on PLRV-infected potato foliage, although these individuals had lower fecundity and shorter longevity when fed virus-infected foliage in comparison with the ones that ate uninfected foliage (Boiteau and Singh, 1982). On the one hand, enhanced CPB larval growth of the ones reared on PVY-infected plants can be explained by inhibited accumulation of products of defense genes, such as proteinase inhibitors, associated with antifeedant properties of potato (Petek et al., 2014). On the other hand, colonization of PVY-infected plants, which had decreased growth, nutritional quality, and ability to regenerate damage caused by beetles, involves certain risks. Booth and Alyokhin (2016) suggested that there may be the strong selection pressure for choosing PVY-uninfected plants. Kersch-Becker and Thaler (2014) showed that the performance of non-vector herbivores *Trichoplusia ni* and *L. decemlineata* positively correlated with the strength of salicylate induction in tomato plants under the influence of PVY.

Thus, plants are exposed to various harmful organisms simultaneously. In this regard, plant protection requires an ever-growing volume of chemical pesticides annually. Unregulated control measures lead to escalation of anthropogenic negative impact on the environment. In addition, arising pathogens and pests resistant to chemicals overcomes protection measures. The search of environmentally safe biocontrol agents, based on beneficent microorganisms that combine diversified biocidal activities against common pathogens and pests and ability to prime immune reactions in plants via stimulation of specific signaling cascades, is of great interest. Some of the beneficial and heterogeneous group plant growth-promoting bacteria (PGPB) able to exist in internal plant tissues and called “endophytes” have drawn increasingly greater attention from researchers and manufacturers of biocontrol agents and biofertilizers (Li et al., 2015; Mishra et al., 2015; Maksimov et al., 2018). Bacterial endophytes have an advantage over bacteria inhabiting the rhizo- or phyllosphere, since living within a plant’s tissues represents an opportunity to be in constant “contact” with the plant’s cells, and endophytes become more integrated in plant metabolism than rhyzo- and phyllospheric microorganisms. PGPB strains show fungicidal (Aydi Ben Abdallah et al., 2016), insecticidal (Sorokan et al., 2016; Yang et al., 2017), and growth-promoting activities (Sansinenea, 2019); synthesize antibiotics and biosurfactants (Maksimov et al., 2020); and induce systemic resistance in plants against pathogens and pests (Rashid and Chung, 2017). The entomopathogenic capacity of *Bacillus thuringiensis* strains to different orders of pests (Diptera, Lepidoptera, Hymenoptera, Coleoptera, Orthoptera, Hemiptera, etc.) involves synthesizing crystalline proteins that have insecticidal activity when ingested by a susceptible host (Domínguez-Arrizabalaga et al., 2019), and now the ability of *B. thuringiensis* strains to invade plant internal tissues is of great interest, as well as insectotoxic properties of endophytic *Bacillus subtilis* strains.

These diverse effects allow the use of PGPB for protection against multiple environmental factors. Thus, *B. thuringiensis* strains can be applied to protect tea plants from both insect and mite pests (Idris et al., 2020). The multifunctional protective effect of endophytic strain *B. subtilis* 26DCry expressing insectotoxic Cry1Ia protein and surfactin against both aphids and pathogens on wheat plants was shown previously (Maksimov et al., 2020). It also should be noted that Bouizgarne (2012) assumed data on plant protection and suggested that in some cases the treatment of plants with endophytes was more advantageous than the cultivation of transgenic plant varieties resistant to viruses. Implementing manufactured plant microbiomes with PGPB that are capable to release antiviral compounds in plants and to prime mechanisms of plant resistance to viral pathogens can become a challenging alternative to chemical pesticides and transgenic plants (Maksimov et al., 2019).

Secretion of enzymes including RNases, which participate in mobilization of organic phosphates, is one of the mechanisms of bacterial adaptation to changing environmental conditions. The ability of *Bacillus pumilus*, *Bacillus amyloliquefaciens*, and *Bacillus licheniformis* to produce extracellular RNases (binases, baRNases, and baliphases, respectively) is actively investigated (Ulyanova et al., 2016; Ilinskaya et al., 2018). Low concentrations of RNases stimulate plant growth and resistance to a broad spectrum of stress factors, and high levels of them show antiviral properties by destroying viral RNA. Thus, all *Pantoea*, *Cronobacter*, *Microbacterium*, and *Staphylococcus* isolates, which originate from Cucurbitaceae plants produce nucleases, as well as 73% of *Bacillus*, 30% of Enterobacteriaceae, and 27% of *Paenibacillus* isolates (Khalaf and Raizada, 2018). *Pseudomonas putida* A3 (Yang et al., 2012) and *B. pumilus* 7P/3-19 (Sharipova et al., 2015) were shown to cleave viral particles in the juice from TMV-infected tobacco plants. The strong positive correlation was shown between the RNase activity in different potato varieties and their resistance to PVX, PVY, PVM, and PVS (Trifonova et al., 2018). Expression of RNase gene PAC1 from *Schizosaccharomyces pombe* in soybean plants led to a significantly higher level of uninfected with prevalent in soybean-growing regions of China soybean mosaic virus (SMV) SC3 strain under the field conditions, compared with the areas where non-transformed plants were grown (Yang et al., 2019). Plants of *Nicotiana benthamiana* containing genetically engineered CRISPR/Cas13a cassette that included class 2 type VI-A RNase capable to recognize and cleave single-stranded RNA were highly resistant to turnip mosaic virus (TuMV) (Aman et al., 2018). Complete resistance to tomato leaf curl virus infection was shown in one third of the transgenic tobacco clones expressing baRNase gene from *B. amyloliquefaciens* (Pakniat-Jahromy et al., 2010). The data shown above tend to favor the view that *Bacillus* can protect plants against viral diseases by affecting phytopathogens, nematodes, and insects, which are vectors of viral particles. At the same time, endophytic *Bacillus* producing RNases can cleave viral particles directly in plant tissues.

In this context, the aim of our work was the investigation of the influence of endophytic RNase-producing *Bacillus* spp. strains on viral and insect spread and potato productivity under the field conditions.

## Materials and Methods

### Bacterial Strains

Bacterial strains *Bacillus subtilis* 26D, *Bacillus thuringiensis* var. *thuringiensis* B-5689, *B. subtilis* 11VM, *B. thuringiensis* var. *kurstaki* B-535, and *B. thuringiensis* var. *kurstaki* B-6066 were provided by the limited liability company Bashincom (Russia). Isolates *B.* sp. STL7 and *Enterobacter* sp. BC-8 were obtained from surface-sterilized intact and CPB-damaged potato leaves, respectively. *Bacillus* sp. TS2 was obtained from surface-sterilized leaves of *Triticum aestivum* L. All isolates were collected from plants that were grown on the territory of Iglinsky District (54°50.48′94.0″N; 56°26.46′09.0″E) of the Republic of Bashkortostan (Russia). All isolates are held in the collection of the Laboratory of Biochemistry of Plant Immunity, Institute of Biochemistry and Genetics, Ufa Federal Research Center RAS^1^.

Selected isolates were characterized through its biochemical and physiological properties according to *Bergey’s Manual of Systematic Bacteriology* (Table 1). Biocontrol agent Bitoksibacilline (BTB) (Sibbiopharm, Russia) on the base of *B. thuringiensis* var. *thuringiensis* BtH_1_98 was used as a positive control. Sequencing of 16S RNA gene fragments of isolates *Bacillus* sp. STL7 (GenBank: MT613864), *Bacillus* sp. TS2 (GenBank: MT605808), and *Enterobacter hormaechei* BC-8 (GenBank: MT605809) was carried out using NovaSeq 6000 Sequencing System (Illumina, United States), with NovaSeq 6000 SP Reagent Kits (Illumina, United States). Nucleotide sequence analysis was presented by using international GenBank database. Bacterial culture was cultivated on lysogeny broth (LB) basal medium (0.5 g/L of NaCl) in TC 1/20 chamber (SPU, Russia) at a temperature 28°C. Sixteen-hour cultures were used for endophytic properties, antiviral activity, and influence on potato productivity estimation.

**TABLE 1:** Biochemical and morphological characterization of bacterial isolates.

Properties	*Bacillus* sp. TS2	*Bacillus* sp. STL7	*Enterobacter* sp. BC-8
Plant	*Triticum aestivum*	*Solanum tuberosum*	*S. tuberosum*
Organ	Stem	Leaves	Leaves
Cell morphology	Rod	Rod	Rod
Gram’s reaction	+	+	–
Spore forming	+	+	–
Grow at 4°C	–	–	–
Grow at 50°C	–	–	–
Grow at 60°C	–	–	–
Anaerobic conditions	–	–	+
Amylase	+	+	–
Protease	+	+	+
Gelatinase	+	–	–
Lipase	+	–	–
Catalase	+	+	+
RNase	+	+	–
Acid from glucose	+	+	+
Gas from glucose	–	–	+
Acid from sacharose	+	+	+
Acid from mannitol	+	+	+
Acid from lactose	–	+	–
Growth on Simmons’s medium (citrate)	+	+	+
Urea hydrolysis	+	–	–
Voges–Proskauer	+	+	+
NH_3_ production	–	–	+
Indole production	–	–	–
H_2_S production	–	–	–
Pigment	–	–	–

### Estimation of Ribonuclease Activity

Quantitative estimation of extracellular RNase activity in liquid culture medium was carried out according to the modified method of Sokurenko et al. (2016). Bacteria strains and isolates were grown on LP medium (low phosphate peptone, 2%; glucose, 1%; Na_2_HPO_4_, 0.04%; CaCl_2_, 0.01%; MgSO_4_^∗^7H_2_O, 0.03%; MnSO_4_, 0.01%; NaCl, 0.3%, 120 μg/ml of phosphorus). Bacteria were cultivated at 37°Ñ using a laboratory shaker ES-20 with oscillation intensity of 120 rpm (Biosan, Lithuania). Culture growth was measured spectrophotometrically at 590 nm and expressed as optical density units (OD590). When density of bacteria reached 10^8^ cells/ml cultures were centrifuged for 15 min at 8,000 *g* in a 5415R centrifuge (Eppendorf, Germany). Potato leaves were homogenized in sterile bags in 1 ml of 0.05 M tris–HCl buffer (pH 8.5) using blender BagMixer 400W (Interscience, France) and incubated for 60 min at 4°C. The homogenates were spun off for 15 min at 8,000 *g* in a 5415R centrifuge (Eppendorf, Germany). pH meter HI 83141 (Hanna Instruments, Romania) was used for pH measurement. Twenty microliters of the homogenates was added to 1.98 ml of 50 μg/ml of torula yeast RNA solution in 0.05 M of tris–HCl buffer (pH 8.5) (Sigma, United States) and kept at 25°C for 1 h, and its absorbance was measured at 260 nm relative to the control (mixture reaction without leaf extract or bacterial medium) at 260 nm on an LLG-uniSPEC 2 spectrophotometer (LLG, Germany). The unit of nuclease activity was taken as the amount of enzyme causing an increase in adsorption by 1.0 optical unit at 260 nm for 1 h at 25°C (Ilinskaya et al., 1996). RNase activity was expressed as units/min⋅ml of liquid medium (extracellular *Bacillus* RNase activity) or units/min⋅mg of protein (RNase activity in plants). Protein concentration in plants was measured using Bradford assay.

### Endophytic Properties

Endophytic content of the tested strains was evaluated by counting the colony-forming units (CFU) of microorganisms in plant tissues 7 days after inoculation of sterile test tube potato plants (Udacha variety) cultivated for 25 days at 16 h illumination (Osram L 36W/77 lamps, Germany) in the KS200 climate chamber (SPU, Russia) on the agarose Murashige–Skoog medium. At least 20 plants were inoculated with 5 ml of each strain (or isolate) suspension (10^8^ cells/ml), which were grown on LB medium. For CFU estimation, 100 mg samples of each individual experimental plant were superficially sterilized in the following order: 70% ethanol (1 min)→0.1% diacide-1 (3 min)→distilled water. The samples were homogenized in sterile bags using BagMixer 400 W blender (Interscience, France) with 2 ml of sterile water added. Two consecutive 10-fold dilutions of the resultant homogenate were then performed. Aliquots (100 μl) were spread over the surface of potato-glucose agar by a microbiological loop until complete drying. Petri dishes were then incubated at 28°C in the TS-1/20 SPU thermostat (Smolensk SKTB SPU, Russia) for 24 h. CFU were counted in the second and third dilutions, and their number was recalculated per 1 g of plant wet weight.

### Field Experiment Design

The study was carried out at the experimental fields of Ufa Federal Science Center RAS (Birsk Experimental Station, 55°25′47.4″N 55°35′49.9″E) during the 2019 and 2020 cropping seasons. There was relatively high temperature at the beginning of the growth season in 2019 and 2020 (Table 2). In 2020, there was a high mean monthly temperature in July compared with the long-term average^2^. An average for 2 years’ data is presented in the figures.

**TABLE 2:** Temperature regime in 2019–2020 cropping seasons.

	Mean monthly temperature,°C		
	May	June	July
2019	14.91	17.39	18.05
2020	13.87	15.83	20.1
Average (2004–2019)	13.3	16.7	18.5

Fields were located on gray forest soils (northern forest steppe). The soils were not water logged, and the texture was sandy loam. The humus content was 3.5–4.0%, and pH of soil was 5.5–6.5. Potato rotation system included wheat and triticale. Seed tubers (elite grade, state standard 33996-201) of the original Udacha variety under study were provided by the Branch of the Federal State Institution “Rosselkhoztsentr” in the Republic of Bashkortostan [certificates 104 105 E1 0115-19 (2019 year), 002 001 E1 0454-20 (2020 year)]. The presence of PVY, PVS, and PVM was not detected.

Tubers were planted at 5 cm depth in rows with 75 cm distance; the interval between tubers in a row was about 30 cm. The planting density was 45,000 tubers per ha. Planting time was the 13th of May. Grimme GL 34T (GRIMME-Rus, Russia) seeding machine was used. The speed of planting was 3 km/ha. Tubers were planted in three repeats of 40 plants per plot for each variant. Three plots were used as replicates for each treatment as well as for the untreated control treatment (water spraying). Two-week seedlings were sprayed with different strains of *Bacillus* suspensions (10^6^ cells/plant) or BTB (50 g/10 L of water, 100 ml/plant). The concentration of the inoculum was determined spectrophotometrically at 600 nm on BioSpec-Mini spectrophotometer (Shimadzu, Japan). Spraying was duplicated after flowering in the same manner.

We observed the density of CPB population on 0, 7, 17, and 31 days in 2019 after the first treatment and on 0, 8, 17, and 29 days after the first treatment in 2020. Season average number (data pooled over the experimental period) (± SE) of CPB egg clusters, early instar larvae (L1 + L2), and last instar larvae (L3 + L4) per plant according to different treatments in 2019–2020 was counted. Defoliation caused by CPB was visually evaluated based on a percentage ranking system; 100% corresponds to complete defoliation, and 0 represents no feeding damage on the 31st day after treatment. Leaves of 30 plants per variant were tested on virus presence and RNase activity on the 17th [distributional of immunofluorescence assay (IFA)-detected viral particles in plants was found to be statistically significant, but visual symptoms of diseases were sporadic] and the 31st day (2019) or 29th day (2020) after the first treatment (visual symptoms became significant on all treated plots). Plants adjacent to free spaces between plots were not examined to avoid edge effect.

Potato tubers were harvested on the 65th (2019 year) and 60th (2020 year) days after the first treatment. On the day of harvest, the tubers were classified into three fractions—small (tubers < 50 g), seed (tubers between 50 and 80 g), and large (tubers > 80 g)—and weighed separately. Data on the productivity of potatoes were prepared according to Bakhvalova et al. (2015).

### Double Antibody Sandwich–ELISA

Direct double antibody sandwich (DAS) ELISA was used with PVM, PVS, and PVY DAS-ELISA Complete kits (Bioreba, Switzerland). Specific rabbit IgG was diluted 1:1,000 in the coating buffer; 200 μl of diluted IgG to each well of Nunc MaxiSorp F96 microtiter plates was pipetted and stored at 4°C overnight. Leaf samples were homogenized in the extraction buffer “General” (DAS-ELISA Complete kits, Bioreba, Switzerland) at a ratio of 1:20 (w/v); 200 μl of the plant extract was added to each well and incubated for 16 h at 4°C. Conjugate of rabbit IgG (anti-potato viruses) with alkaline phosphatase 1:1,000 in conjugate buffer was added and incubated at 30°C for 5 h; after this, 200 μl of the substrate pNPP (*para*-nitrophenylphosphate) in substrate buffer pH 9.8 (DAS-ELISA Complete kits, Bioreba, Switzerland) (1 mg/ml) were added. After 30 min (20–25°C in the dark), optical density was measured at 405/492 nm on plate reader Uniplan AIFR-01 (CJSC Picon, Russia).

### Statistics

Laboratory experiments were repeated three times in three replications. Mean values with standard errors (± SE) are given in the figures and tables. Statistical analyses were carried out using Microsoft Excel 2013 for Windows (Microsoft Corporation, United States) and IBM SPSS Statistics 2.0 (IBM Corporation, United States). Differences in parameters under investigation between individual treatments were analyzed with the use of ANOVA. Prior to analysis, each variable was tested for homogeneity of variance, and the data found to be non-homogenous were transformed to log(Y) before ANOVA. Significant differences (*P* ≤ 0.05) between mean values were identified using Tukey’s honestly significant difference (HSD) multiple-range test. Different letters on figures label significant differences between treatments according to Tukey’s HSD multiple-range test at *P* < 0.05. Pearson’s correlation coefficient was used to understand the nature of relationships between individual parameters.

## Results

### Endophytic Properties and Ribonuclease Activity of *Bacillus* Strains

We showed the presence of RNase activity in all *Bacillus* strains and isolates under investigation (Table 3). Isolated from CPB-damaged potato leaves, *Enterobacter* sp. BC-8 did not express any RNase activity. The significant RNase activity was observed in the liquid culture medium of *Bacillus thuringiensis* B-6066, *Bacillus* sp. STL-7, *Bacillus* sp. TS2 and *Bacillus subtilis* 26D, and *B. thuringiensis* B-5689 and minimally in the culture medium of *B. thuringiensis* B-5351.

**TABLE 3:** RNase activity of bacterial strains *in vitro* and its CFU content in internal tissues of potato stems.

Variant/parameter	*Bacillus* sp. TS2	*Bacillus* sp. STL7	*Bacillus thuringiensis* B-6066	*Bacillus subtilis* 11VM	*B. subtilis* 26D	*B. thuringiensis* B-5351	*Enterobacter* sp. BC-8	*B. thuringiensis* B-5689
RNase, OD/min⋅ml of culture medium	6.45 ± 1.03a	5.87 ± 0.96a	6.02 ± 0.86a	3.04 ± 0.55b	3.97 ± 0.77a	1.32 ± 0.08c	0d	2.64 ± 0.04b
Endophyticity, CFU * 10^3^/g of wet mass	120.0 ± 14.2a	3.5 ± 0.1b	90.0 ± 13.4c	90.0 ± 12.3c	350.0 ± 15.6d	70.0 ± 12.3c	0.01 ± 0.001e	3.0 ± 0.11b

*–, none detected. Data represented as mean values ± standard error; Values followed by the same alphabet within a row are not significantly different from each other by Tukey HSD test P < 0.05. CFU, colony-forming units; HSD, honestly significant difference.*

Bacteria *B. subtilis* 26D was found in potato plant tissues in the amount of 350 ^∗^ 10^3^ CFU/g of wet weight. The CFU numbers of *Bacillus* sp. TS2, *B. subtilis* 11VM, and *B. thuringiensis* B-5351 were broadly similar (70–120 ^∗^ 10^3^ CFU/g of wet weight). The ability of *B. thuringiensis* B-5689 and *Bacillus* sp. STL7 was an order of magnitude less than that of other strain showed, and the amount of *Enterobacter* sp. BC-8 was very low, and this strain was used as a negative control in the field experiments.

Thus, it was shown that the strains *B. subtilis* 26D and *Bacillus* sp. TS2 had a greater ability to actively invade and colonize plant tissues as compared with other strains and relatively high RNase activity and when then grown on LP medium cultures were used for inoculation. The influence of RNase activity on endophytic properties of strains was not significant (Table 4).

**TABLE 4:** Statistical analysis of influence of treatment, RNase activity of strains and isolates and endophytic rate of strains and isolates on different parameters of potato plants under the field conditions.

Early period	*F*	*df*	*P*	Latest period	*F*	*df*	*P*
**Treatment**							
RNase activity in plants	6.78	9,590	<0.05	RNase activity in plants	5.07	9,590	<0.05
Eggs per plant	6.88	9,590	<0.05				
Young instar larvae per plant	14.07	9,590	<0.05	Last instar larvae per plant	4.97	9,590	<0.05
				Consumed leaves per plant	4.86	9,590	<0.05
PVY infected plants per plot	4.67	9,50	<0.05	PVY infected plants per plot	6.84	9,50	<0.05
PVS infected plants per plot	5.08	9,50	<0.05	PVS infected plants per plot	4.95	9,50	<0.05
PVM infected plants per plot	4.86	9,50	<0.05	PVM infected plants per plot	12.75	9,50	<0.05
PVY + PVM infected plants per plot	7.25	9,50	<0.05	PVY + PVM infected plants per plot	6.84	9,50	<0.05
PVY + PVS infected plants per plot	5.43	9,50	<0.05	PVY + PVS infected plants per plot	6.36	9,50	<0.05
PVM + PVS infected plants per plot	8.43	9,50	<0.05	PVM + PVS infected plants per plot	4.81	9,50	<0.05
				Viral symptoms per plant	5.34	9,290	<0.05
				Total weight	7.95	9,50	<0.05
				Weight of tubers in fraction = 80 g	8.65	9,50	<0.05
				Weight of tubers in fraction = 50 g	4.32	9,50	>0.05
				Weight of tubers in fraction <50 g	4.44	9,50	<0.05
**RNase activity in culture medium**							
Endophytic properties of bacteria	2.04	3,298	>0.05				
RNase activity in plants	9.07	3,598	<0.05	RNase activity in plants	4.01	3,598	>0.05
Eggs	3.87	3,598	>0.05				
Young instar larvae	7.84	3,598	>0.05	Last instar larvae	7.98	3,598	>0.05
				Consumed leaves per plant	6.34	3,598	>0.05
PVY infected plants per plot	17.27	3,58	<0.05	PVY infected plants per plot	8.89	3,58	<0.05
PVS infected plants per plot	3.76	3,58	>0.05	PVS infected plants per plot	7.46	3,58	>0.05
PVM infected plants per plot	7.84	3,58	>0.05	PVM infected plants per plot	8.03	3,58	>0.05
PVY + PVM infected plants per plot	9.34	3,58	<0.05	PVY + PVM infected plants per plot	8.87	3,58	<0.05
PVY + PVS infected plants per plot	9.43	3,58	<0.05	PVY + PVS infected plants per plot	9.05	3,58	<0.05
PVM + PVS infected plants per plot	8.12	3,58	>0.05	PVM + PVS infected plants per plot	6.84	3,58	>0.05
				Viral symptoms per plant	11.34	3,58	<0.05
				Total weight	7.99	3,58	>0.05
				Weight of tubers in fraction = 80 g	5.78	3,58	>0.05
				Weight of tubers in fraction = 50 g	3.76	3,58	>0.05
				Weight of tubers in fraction <50 g	3.76	3,58	>0.05
**Endophytic rate**							
RNase activity in plants	5.34	4,597	>0.05	RNase activity in plants	4.67	4,597	>0.05
Eggs	6.88	4,597	<0.05				
Young instar larvae	10.97	4,597	<0.05	Last instar larvae	6.97	4,597	<0.05
				Consumed leaves per plant	6.86	4,597	<0.05
PVY infected plants per plot	3.78	4,57	>0.05	PVY infected plants per plot	5.03	4,57	>0.05
PVS infected plants per plot	5.87	4,57	<0.05	PVS infected plants per plot	7.56	4,57	<0.05
PVM infected plants per plot	7.12	4,57	<0.05	PVM infected plants per plot	6.17	4,57	<0.05
PVY + PVM infected plants per plot	3.02	4,57	>0.05	PVY + PVM infected plants per plot	4.57	4,57	>0.05
PVY + PVS infected plants per plot	4.55	4,57	>0.05	PVY + PVS infected plants per plot	1.77	4,57	>0.05
PVM + PVS infected plants per plot	5.14	4,57	>0.05	PVM + PVS infected plants per plot	3.01	4,57	>0.05
				Viral symptoms per plant	5.94	4,597	<0.05
				Total weight	5.78	4,57	<0.05
				Weight of tubers in fraction = 80 g	8.37	4,57	<0.05
				Weight of tubers in fraction = 50 g	3.87	4,57	>0.05
				Weight of tubers in fraction <50 g	5.01	4,57	>0.05

### Colorado Potato Beetle Population

**FIGURE 1:**
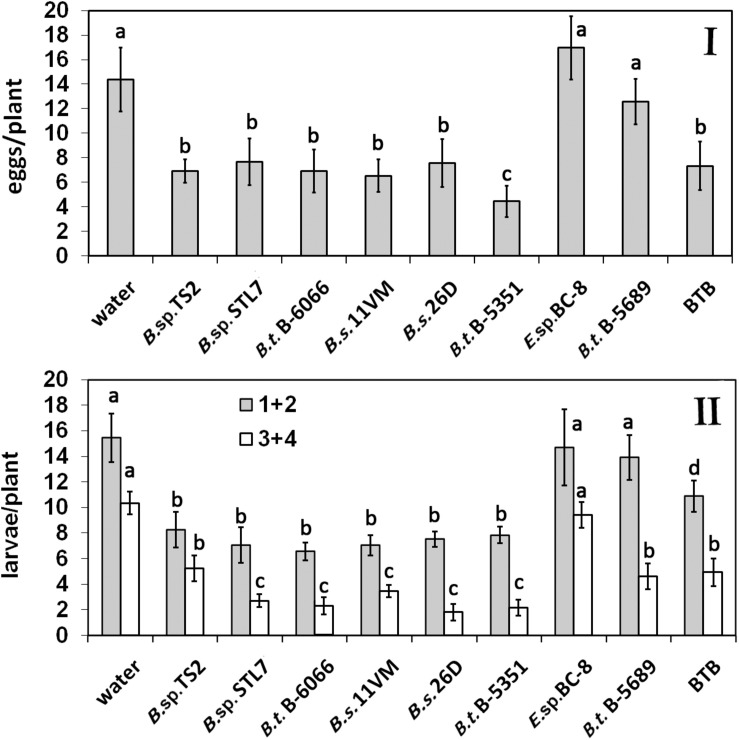
Effect of bacterial agents and Bitoksibacilline (BTB) treatments of potato plants on the total number of eggs **(I)** and larvae **(II)** of Colorado potato beetle (CPB) per plant during the whole seasons of 2019 and 2020 (data pooled over the experimental period) (1 + 2, early instar larvae; 3 + 4, last instar larvae). Data represented as mean values ± standard error; values followed by the same alphabet within a column are not significantly different from each other according to Tukey’s honestly significant difference (HSD) multiple-range test at *P* < 0.05.

The average number of CPB eggs per plant ranged from 14.0 ± 4.5 and 15.4 ± 3.9 on water-treated and *Enterobacter* sp. BC-8 plants, respectively, to 4.6 ± 2.2 on *B. thuringiensis* B-5351-treated plants (Figure 1I). A relatively low rate of egg clusters was observed on plants under the influence of *Bacillus* sp. STL7 and TS2, *B. subtilis* 11VM and 26D, and *B. thuringiensis* B-6066. Their effectiveness was similar to that of commercial biocontrol agent BTB (Figure 1I).

The average seasonal number of CPB early instar larvae per plant without endophytes was 15.8 ± 1.23 (Figure 1II). There were no statistically significant differences among water, *Enterobacter* sp. BC-8, and *B. thuringiensis* B-5689 treatments (*P* > 0.05). A significantly lower number of early instar larvae was found on plots treated with *B. thuringiensis* B-6066 and *B. thuringiensis* B-5351, *B. subtilis* strains under investigation, and *B.* sp. STL7 and *Bacillus* sp. TS2.

The number of last instar larvae on water-treated plants was 10.9 ± 0.64 per plant. *Bacillus* sp. STL7, *B. subtilis* 11VM, *B. subtilis* 26D, *B. thuringiensis* B-5689, *B. thuringiensis* B-6066, and BTB treatment gave a low number of last instar larvae (one third of control number). The amount of last instar larvae did not differ significantly from each other in plants that were under the influence of *Bacillus* sp. TS2, *B. thuringiensis* B-5689, and BTB accounted for half of this parameter on water-treated ones. On *Enterobacter* sp. BC-8 cell-treated plots, a higher number of last instar larvae per plant as compared with *Bacillus* spp.-treated plots were observed.

### Potato Virus Incidence

**FIGURE 2:**
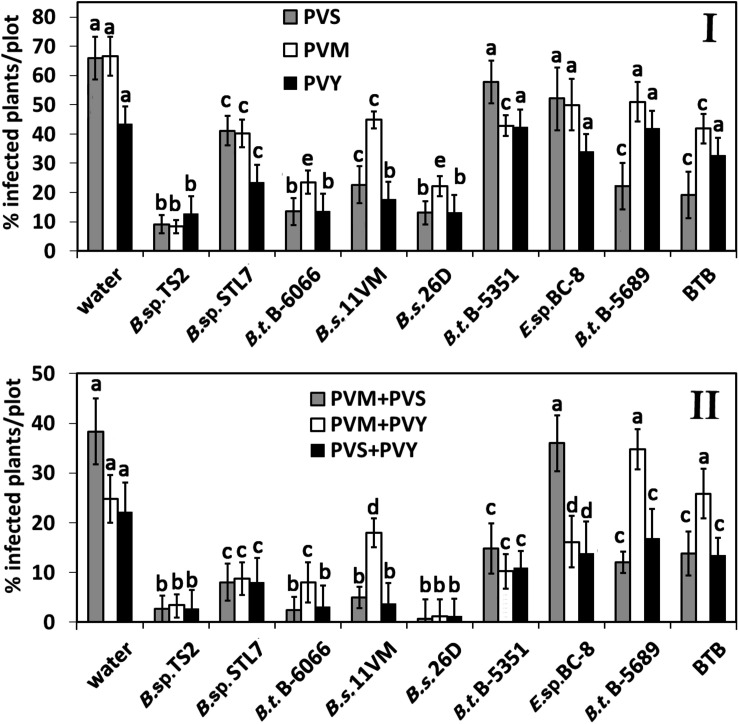
Effect of bacterial agents and Bitoksibacilline (BTB) treatments of potato plants on the prevalence of potato virus Y (PVY), potato virus M (PVM), and potato virus S (PVS) on potato plots on the 17th day after treatment: percentage of samples that tested positively for each virus **(I)** and samples that tested positive for two viruses simultaneously **(II)**. Data represented as mean values ± standard error; values followed by the same alphabet within a column are not significantly different from each other according to Tukey’s honestly significant difference (HSD) multiple-range test at *P* < 0.05.

Under the field conditions, a significant reduction of PVS incidence was recorded in plants treated with *Bacillus* sp. TS2, *B. subtilis* 26D, *B. thuringiensis* B-6066 (*P* < 0.01 in all cases) on the 17th day after the first treatment. The effect of another *Bacillus* spp. strains and isolate on PVS prevalence decrease was less severe but significant (*P* < 0.005) as compared with the water, *B. thuringiensis* B-5351, or *Enterobacter* sp. BC-8 treatments (Figure 2I). PVM was found in about 70% of potato plants, treated with water, *B. thuringiensis* B-5689, *Enterobacter* sp. BC-8, and BTB. The lowest percentage of PVM-infected plants was observed on *Bacillus* sp. TS2-treated plots (8.3%). *B. subtilis* 26D and *B. thuringiensis* B-6066 decreased the incidence of PVM-positive plants by about 45% as compared with the water treatment. *B.* sp. STL7, *B. subtilis* 11VM, and *B. thuringiensis* B-5351 treatments were less effective, but decrease of viral incidence was significant in comparison with that of water-treated plots (*P* < 0.05 in all cases) and was as much as 15%. Decrease of PVY prevalence was observed on plots that were sprayed with *Bacillus* sp. TS2, *Bacillus* sp. STL7, *B. subtilis* 11VM, *B. subtilis* 26D, and *B. thuringiensis* B-6066 (*P* < 0.01 in all cases). It is important that *Bacillus* sp. TS2, *B. subtilis* 26D, and *B. thuringiensis* B-6066 contributed to decrease of prevalence rate of all viruses under investigation, and the incidence of plants that tested positive for two viruses simultaneously in these cases was less than 10%; then in water-treated plots, this rate was more than 35% (PVM + PVS) and more than 25% (PVM + PVY and PVS + PVY) (Figure 2II).

A relatively low rate of mixed infections was observed in plots treated with *B.* sp. STL7 (about 10%), *B. subtilis* 11VM (about 5% of plants infected with PVS + PVM and PVS + PVY), and *B. thuringiensis* B-5351 (about 15% of plants tested positive on two viruses simultaneously).

**FIGURE 3:**
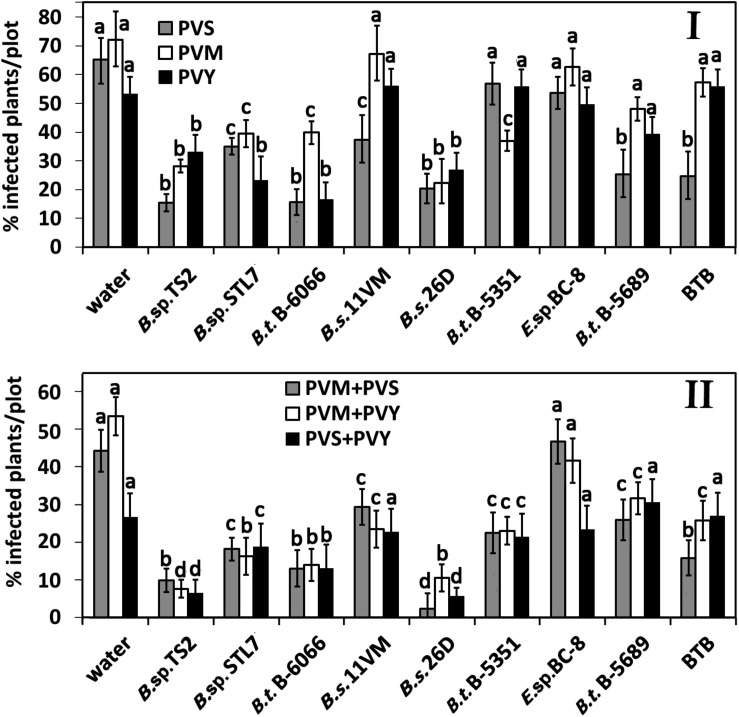
Effect of bacterial agents and Bitoksibacilline (BTB) treatments of potato plants on the prevalence of potato virus Y (PVY), potato virus M (PVM), and potato virus S (PVS) in potato plots on the 31st day (2019) and 29th day (2020) after treatment: percentage of samples that tested positively for each virus **(I)** and samples that tested positive for two viruses simultaneously **(II)**. Data represented as mean values ± standard error; values followed by the same alphabet within a column are not significantly different from each other according to Tukey’s honestly significant difference (HSD) multiple-range test at *P* < 0.05.

On the 31st (2019) and 29th (2020) days after first treatment, percentage of infected plants was not increased in plots treated with water, *B. thuringiensis* B-5351, *B. thuringiensis* B-5689, *Enterobacter* sp. BC-8, BTB, and *B.* sp. STL7 (Figure 3I). In *Bacillus* sp. TS2- and *B. subtilis* 26D-treated plots, the number of virus-infected plants increased by 10–15% compared with the previous measure. The number of PVM and PVY infected plants on *B. subtilis* 11VM-treated plots became the same as that of control plots. On BTB-treated plots, the number of PVM-infected plants had risen to those of control ones.

The rise of the number of plants tested positively on more than a single virus was observed on the 31st day after treatment (Figure 3II). A relatively low rate of mixed infections was observed in plots treated with *B. thuringiensis* B-6066, *B. subtilis* 26D, and *Bacillus* sp. TS2 (about 8–12% of plants in all cases); *Bacillus* sp. STL7 (about 15% of plants); and *B. subtilis* 11VM (increase of number of PVM + PVS- and PVS + PVY-infected plants). Importantly, the treatment with *B. subtilis* 26D led to the least number of plants positive as tested on PVS mixed infections.

Thus, treatments of plants with bacterial strains *B. subtilis* 11VM, *Enterobacter* sp. BC-8, *B. thuringiensis* B-5689, and bioinsecticide BTB were least efficient against the most damaging viral disease that caused PVY and mixed infection of PVY (Figure 3II).

### Ribonuclease Activity in *Bacillus*-Treated Plants Under the Field Conditions

**FIGURE 4:**
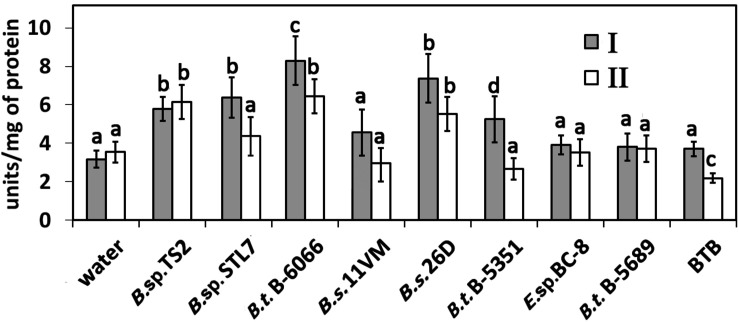
RNase activity in potato plants treated with bacterial strains and Bitoksibacilline (BTB) on the 17th day **(I)** and the 31st day after treatment **(II)**. Data represented as mean values ± standard error; values followed by the same alphabet within a column are not significantly different from each other according to Tukey’s honestly significant difference (HSD) multiple-range test at *P* < 0.05.

The RNase activity rate in water-sprayed potato plants was about 3.5–4 units/mg of protein (Figure 4). Treatment of plants with *Bacillus* sp. TS2, *B. subtilis* 26D, and *B. thuringiensis* B-6066 caused significant increase of RNase activity on both the 17th and 31st (2019) and 29th (2020) days after first treatment (*P* < 0.005). *Bacillus* sp. STL7 and *B. thuringiensis* B-5351 promoted RNase activity on the 17th day after first treatment almost equally, but this parameter became equal to the control ones subsequently. RNase activity in plants treated with other strains under investigation was equal to that estimated in water-treated ones.

### Defoliation and Viral Disease Symptoms

**FIGURE 5:**
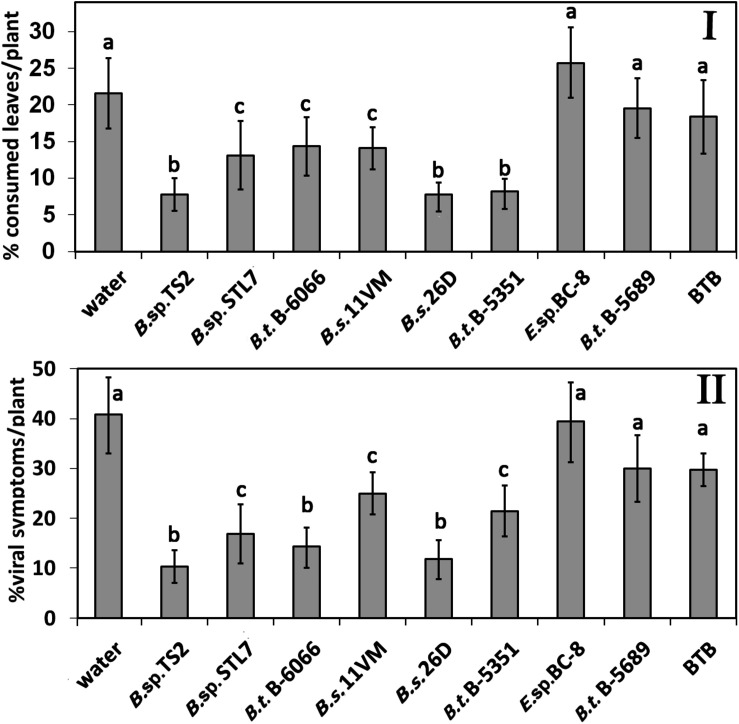
Defoliation of potato plants caused by *Leptinotarsa decemlineata* **(I)** and percentage of viral diseases damaged leaves **(II)** of potato plants treated with bacterial agents and Bitoksibacilline (BTB). Data represented as mean values ± standard error; values followed by the same alphabet within a column are not significantly different from each other according to Tukey’s honestly significant difference (HSD) multiple-range test at *P* < 0.05.

Defoliation caused by *Leptinotarsa decemlineata* was minimal on plots treated with *Bacillus* sp. TS2, *B. thuringiensis* B-5351, and *B. subtilis* 26D. In these cases, it was almost one third of this parameter in water-treated plants (*P* < 0.01) (Figure 5I). Significant reduction of the defoliation level was observed on plants, treated with *B. thuringiensis* B-6066, *Bacillus* sp. STL7, and *B. subtilis* 11VM (*P* < 0.05). The influence of *Enterobacter* sp. BC-8, *B. thuringiensis* B-5689, and biocontrol agent BTB did not show any significant influence on defoliation.

Viral disease symptoms including leaf deformation (crinkling) and size reduction with shiny appearance and yellow mottling that were caused by PVY or complex of PVY and PVM and/or PVS or chlorosis and mottling (complex of PVM and PVS) were estimated visually. Leaves with viral diseases signs constituted about a quarter of the leaves of entire plants treated with water, *Enterobacter* sp. BC-8, *B. thuringiensis* B-5351, and BTB. Disease severity significantly (*P* < 0.05) decreased under the influence of *B. thuringiensis* B-6066, *B.* sp. STL7, and *B. subtilis* 11VM (Figure 5II). In plants treated with *Bacillus* sp. TS2, *B. subtilis* 26D, and *B. thuringiensis* B-5689, the lowest rate of leaves with disease symptoms was achieved (*P* < 0.01).

### Potato Yield Structure

**FIGURE 6:**
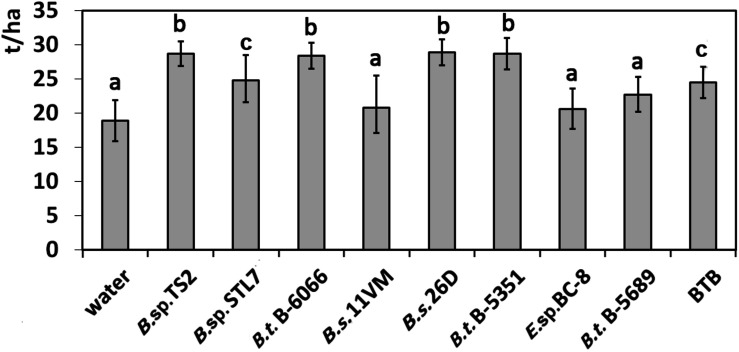
Effect of bacterial agents and Bitoksibacilline (BTB) treatments of potato plants on the total potato yield. Data represented as mean values ± standard error; values followed by the same alphabet within a column are not significantly different from each other according to Tukey’s honestly significant difference (HSD) multiple-range test at *P* < 0.05.

Among treatments with water, *Enterobacter* sp. BC-8, *B. subtilis* 11VM, and *B. thuringiensis* B-5689, we did not establish significant differences in productivity (Figure 6). Slight increase of the yield was observed under the influence of *Bacillus* sp. STL7 and BTB. *Bacillus* sp. TS2, *B. thuringiensis* B-6066, *B. thuringiensis* B-5351, and *B. subtilis* 26D treatments promoted the highest total yield.

**FIGURE 7:**
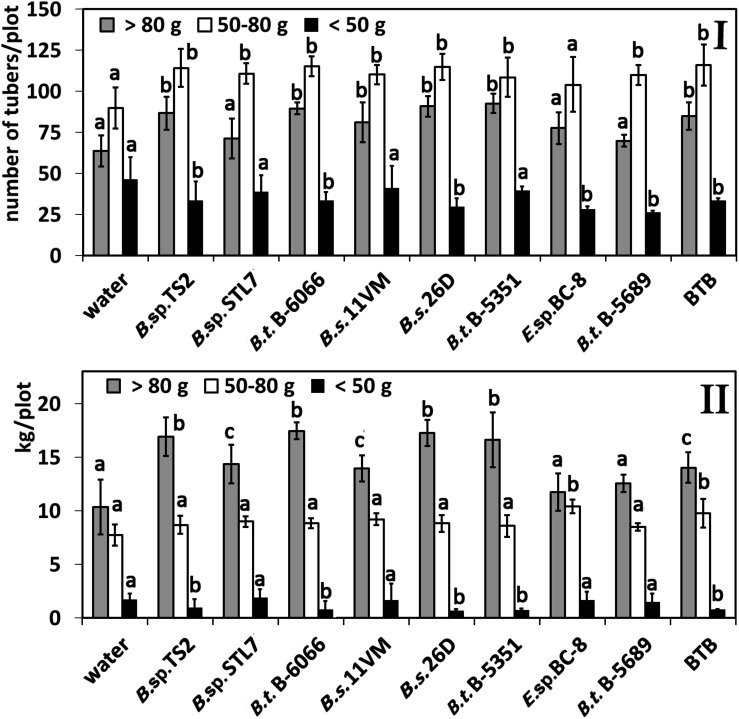
Effect of bacterial agents and Bitoksibacilline (BTB) treatments of potato plants at different fractions. **(I)** Number of tubers in fraction; **(II)** weight of tubers in fractions. Data represented as mean values ± standard error; values followed by the same alphabet within a column are not significantly different from each other according to Tukey’s honestly significant difference (HSD) multiple-range test at *P* < 0.05.

The number of tubers in the fraction > 80 g was increased under the influence of all *Bacillus* spp. strains except *Bacillus* sp. STL7 and *B. thuringiensis* B-5689; in the fraction of 50–80 g, an increased amount was observed under the influence of all *Bacillus* treatments (Figure 7I). A decrease in the number of tubers in the fraction < 50 g under the influence of *Bacillus* sp. TS2, *B. thuringiensis* B-6066, *B. thuringiensis* B-5689, *B. subtilis* 26D, and *Enterobacter* sp. BC-8 was observed.

Treatments with *Bacillus* sp. TS2, *B. thuringiensis* B-6066, *B. thuringiensis* B-5153, and *B. subtilis* 26D had the major weight of large (> 80 g) tubers, while the least total weight of this fraction had tubers from plots that were treated with water, *Enterobacter* sp. BC-8, and *B. thuringiensis* B-5689 (Figure 7II). *Bacillus* sp. STL7, *B. subtilis* 11VM, and BTB promoted the increase of average weight of tubers in this fraction to a lesser degree than other strains under investigation and in accordance with the increase of total yield in these cases. Weight of tubers in the middle fraction was higher than that registered in water-treated plants only in cases of treatments with *Enterobacter* sp. BC-8 and BTB. Treatment of potato plants with cells of *Bacillus* sp. STL7 and *B. thuringiensis* B-5689 and *Enterobacter* sp. BC-8 were not influenced on weight of tubers in the fraction < 50 g in contrast to other strains that decrease this parameter.

## Discussion

Modern agriculture is the foundation stone for food security and one of the major dangers compromising the environment on a global scale. Plants, most especially agricultural species, are constantly falling under diverse biotic impacts, which damage plant organisms and decrease production value and quality. On the one hand, at the moment, the greatest task is improving or maintaining the food quality. On the other hand, it is important to keep damage caused to the environment to a minimum. Viruses cause epiphytotics in all species of cultivated plants, but the control of viral vectors is still a solitary measure to decrease their presence. Thus, there are no currently effective chemical means of plant protection against viruses. Unfortunately, there are not much dominant resistance genes against viruses in cultivated plants. Genome-editing technologies are more efficient to control viral diseases but raise concerns for their safety.

Numerous researches on beneficial microorganisms exist, but there is little information on the ability of biological agents to control the spread of different harmful pathogens and pests which simultaneously influence agricultural plants. And furthermore, some research of the influence of PGPB on the spread of viruses focuses on their impact on insect vectors, such as aphids and whiteflies. Viral infections of plants, however, had a significant effect on behavior of the CPBs. Booth and Alyokhin (2016) showed that CPB larvae and adults predominantly colonized virus-free plants in comparison with PVY-infected plants, since infected plants had poorer growth and reduced ability to sprout novel foliage after CPB damage. The latest can determine strong selection pressure for choosing PVY-free plants. PVY infection decreased plant resistance to non-vector herbivores, *Leptinotarsa decemlineata*, and *Trichoplusia ni* in increasing their growth rates (Kersch-Becker and Thaler, 2014). The development of efficient and durable resistance of plants able to withstand viral attacks represents a major challenge for agrobiology. Currently, the spread of viruses cannot be controlled with chemical pesticides, and microbes that are beneficial to plants can be an alternative due to their ability to synthesize antiviral compounds directly in plant tissues or induce plant defense reactions.

The capability of PGPB to moderate virus spread and reproduction in plants was reported in a great number of studies (Maksimov et al., 2020). Unfortunately, there are only few studies available on the status of these bacterial strains within host plants and their ability to interact with the host and to exist endophytically. Observed under laboratory or greenhouse conditions, the potential of PGPB to improve crop production and increase yields became unexpected and incalculable under different field conditions (Bly et al., 2009). The sensitivity of PGPB strains to the plant defense mechanisms and soil conditions as well as usual flushing with rain limits their ability to colonize the rhizosphere and plant tissues or surfaces and express beneficial influence on host plants (Cakmakçi et al., 2006). The promising approach of agricultural plant protection against viruses involves investigating the composition and role of plant microbiome in order to develop environmentally safe biocontrol agents with diverse beneficial properties (growth-promoting activity, priming of plant immune reactions against pathogens and pests, and direct pesticidal effects) for plant protection against viral diseases and vector and non-vector pests.

In this study, we investigate *Bacillus* spp. strains capable of penetrating internal plant tissues in different degrees. The *Bacillus subtilis* 26D showed the maximal number of CFU in potato plant tissues; *Bacillus thuringiensis* B-5689 showed the minimal number among endophytic strains. *Enterobacter* sp. BC-8 had no endophytic properties. This strain was found in CPB intestines (Sorokan et al., 2019), and its presence in internal plant tissues can partly be explained by CPB attacks. The endophytic rate of bacteria significantly influenced insect population (number of eggs and young larvae per plant) of consumed leaves per plant, PVS and PVM incidence on early and latest stage and viral symptoms on leaves per plant, and plant productivity (weight of tubers in the fraction ≥ 80 g) (see Supplementary Material).

It was found that according to the results of the 2-year experiments between “endophytic level” of the strain, used for treatment and productivity of plants (*r* = 0.745256), weight of tubers in the fraction ≥ 80 g (*r* = 0.764837), number of young larvae per plant (*r* = −0.643567), and PVS incidence (*r* = −0.865972), strong correlations were observed. Thus, the number of cells of endophytic microorganisms in plant tissues itself was not a decisive factor in plant protection against viruses, but, probably, this property allowed PGPB to synthesize their metabolites, in particular RNases and Bt-toxines, inside plant organisms.

A lot of *Bacillus* species produce extracellular high-molecular-weight RNases (Maksimov et al., 2019). It was established that treatment of tobacco plants with 100 μg/mg of RNase from *Bacillus pumilus* directly suppressed the development of PVS and PVM infection and almost completely inhibited PVX infection, as well as decreased the red clover mottle virus (RCMV) particle number in pea plants (Fedorova et al., 2011). *B. pumilus* 7P/3-19 extracellular RNases decreased the spread of RNA viruses RCMV, PVX, and alfalfa mosaic virus in pea plants. The maximum inhibitory effect against viruses under investigation was observed when plants were treated with 100 μg/ml of RNase prior to infection (Sharipova et al., 2015). In this regard, an alternative strategy for protecting plants from viruses can be based on the use of microbial enzymes, for example, extracellular nucleases or proteases of *Bacillus* spp. In our investigation, the strong impact of RNase activity of bacterial agents used for the treatment on PVY incidence (early stage *r* = −0.76322, latest stage *r* = −0.5734), mixed infections PVM + PVY (early stage *r* = −0.8675), and PVY + PVS (early stage *r* = −0.6543) incidence was observed (Table 4).

It is worth noting that the effect of RNase activity of bacteria on total RNase activity in potato plants under the field conditions was significant for early stage of viral spreading, and the level of RNase activity of bacterial strain *in vitro* corresponded to the total level of RNase activity in potato plants (system plant + endophyte). Later, a high level of RNase activity was observed only in plants treated with agents, which demonstrates high RNase activity and pronounced ability to establish endophytic relations with host plant simultaneously (*Bacillus* sp. TS2, *B. thuringiensis* B-6066, and *B. subtilis* 26D). This fact probably increased plant resistance to viruses. Thus, for buckwheat varieties Roksolana and Kara-Dag with different resistance to the buckwheat burn virus (BBV), a positive correlation between resistance to virus and RNase activity was shown (Sindarovska et al., 2014). Trifonova et al. (2018) have proposed to use the level of RNase activity in potato leaves as a selective marker for resistance to viruses.

Since endophytic strains producing RNases inhibited the spread of viruses and affected viral symptom expression, bacterial strains combining a high endophytic rate and high RNase activity, such as *Bacillus* sp. TS2 and *B. subtilis* 26D, can be used for biocontrol agents development. Hameed et al. (2014) concluded that multiple viral infections (for example, widespread PVS + PVY and PVM + PVY joint infections) cause dramatic escalation of plant damage as compared with a single infection. Under the influence of *Bacillus* sp. TS2, and *B. subtilis* 26D abundance of plants positively tested on more than one virus tend toward zero, and these bacterial agents should be considered as effective means for plant protection against viral diseases.

In our investigation, percentage of defoliation caused by CPB correlated with disease symptoms severity (*r* = 0.6771). It was found previously that pea enation mosaic virus (PEMV)-infected pea plants experienced more defoliation from non-vector pea leaf weevil, *Sitona lineatus*, than uninfected plants. In turn, vector of PEMV, pea aphid *Acyrthosiphon pisum*, prevalently affected plants that were previously being damaged by *S. lineatus*. Weevil herbivory forced PEMV titer in infected plants (Chisholm et al., 2018). Su et al. (2020) found that non-vector *Tetranychus urticae* infestation promoted vector *Bemisia tabaci* feeding of *Solanum lycopersicum* plants, thereby increasing tomato yellow leaf curl virus (TYLCV) transmission to tomato plants.

*Bacillus* sp. TS2, *B. thuringiensis* B-5351, and *B. subtilis* 26D significantly decreased defoliation caused by CPB; i.e., they showed antifeedant effect on CPB when they were used for potato plants treatment as well as attractiveness of plants for oviposition. It is particularly significant that *B. thuringiensis* B-5689 provided for a one-third increase in the mortality of *L. decemlineata* larvae as if under the influence *of B. subtilis* 26D under the laboratory conditions (Sorokan et al., 2018) but had no significant impact on pest population density in the field, probably due to its low ability to invade internal plant tissues. We found that endophytic strains that showed insecticidal activity—*B. thuringiensis* B-5351 and *B. thuringiensis* B-6066 (Sorokan et al., 2019), synthesizing Cry-protein, and *B. subtilis* 26D disturbing CPB microbiota (Sorokan et al., 2016)—effectively decreased abundance of *L. decemlineata* eggs, and young and old larvae and the defoliation level. Thus, there were steady and substantial limited amounts of plants infected with viruses and disease severity on plots treated with bacteria, which combine pronounced entophytic properties, RNase activity, and previously established insecticidal activity.

The population density of CPB in Laznik et al. (2010) field experience increased since the beginning of the vegetation season, and in the middle of cultivation period, damage caused by CPB was the most serious. Weight and amount of small tubers harvested from plants that were less damaged (thiamethoxam treatment) were smaller, while the mass of large tubers was higher than from severely damaged plants. Thus, defoliation of potato plants influenced the tuber developments in such a way that potato plants produced smaller tubers that were unable to grow in size. In our investigation, treatment with *Bacillus* sp. TS2, *B. subtilis* 26D, *B. thuringiensis* B-6066, and *B. thuringiensis* B-5351 increased the total yield and weight of large tubers (≥ 80 g) harvested per plot. The number of tubers in this class was equally increased compared with that of control ones in all cases except *Enterobacter* sp. BC-8 and *B. thuringiensis* B-5689. The increase of total yield, in particular, weight of tubers in the fraction ≥ 80 g, was correlated depending on the range of bacteria in the internal tissues (*r* = 0.77353).

Amount and the mass of medium (50–80 g) tubers were the same in plots under the influence of all *Bacillus* under investigation. In the cases of plants treated with *B. subtilis* 26D, *B.* sp. TS2, *B. thuringiensis* B-5351, and *B. thuringiensis* B-6066, which increased the weight of large tubers, the formation of small tubers was lower than of control plots. Similar results were reported by Bakhvalova et al. (2015), who showed that the biomass of potato tubers was increased under the influence of *B. thuringiensis* H10 strain due to the enhancement of quantity and weight of tubers in ≥ 80-g class, and distribution of Rhizoctonia on potato stolons decreased almost twice. Soil drenching with *B. subtilis* KU936344, KU936345, and KU936341 strains led to the higher yield of market-grade potato tubers and less unfit for trading tubers, even when compared with plots treated with fungicide Mancozeb (Kumbar et al., 2019). It is important that, in our experiments, high yield was obtained under the influence of strains that combine endophytic properties, RNase activity, and antifeedant effect on the pest.

## Conclusion

Thus, the reinforcement of agroecosystems with endophytic microorganisms that can produce RNases directly in plant tissues is a promising strategy for the advancement of virus management. Results of our research showed that endophytic *Bacillus* sp. TS2, *B. subtilis* 26D, and *B. thuringiensis* B-5351 were effective biological agents in the control of CPB and viral diseases of potato. These strains can be used for the development of multifunctional biocontrol agents (insecticide + viricide), which can be more effective than modern mono-active chemical pesticides.

## Data Availability Statement

The datasets presented in this article are not readily available because: there is not any restrictions. Requests to access the datasets should be directed to fourtyanns@googlemail.com.

## Author Contributions

AS designed and conducted the study, harvested the samples, collected the data, and wrote the manuscript. EC, GBu, SV, and ES conducted the study and collected the data. SR, VA, GBe, and IM harvested the samples and conducted field experiments. RK polished the manuscript. IM proposed the research and managed the funding. All authors contributed to the article and approved the submitted version.

## Conflict of Interest

The authors declare that the research was conducted in the absence of any commercial or financial relationships that could be construed as a potential conflict of interest.

## Footnotes

1 http://ibg.anrb.ru/wp-content/uploads/2019/04/Katalog-endofit.doc

2 http://weatherarchive.ru/Temperature/Birsk/

## Abbreviations

BTB: Bitoksibacilline
CFU: colony-forming units
CPB: Colorado potato beetle
LB: lysogeny broth
LP: low phosphorus medium
PGPB: plant growth-promoting bacteria
PVM: potato virus M
PVS: potato virus S
PVY: potato virus Y.
