# Supplementary material for: Endophytic Bacillus spp. as a Prospective Biological Tool for Control of Viral Diseases and Non-vector Leptinotarsa decemlineata Say. in Solanum tuberosum L
Source: Front Microbiol. 2020 Oct 15;11:569457. doi: 10.3389/fmicb.2020.569457 (PMC7593271; doi:10.3389/fmicb.2020.569457)
Supplement: Supplementary file 1 [file Data_Sheet_1.DOCX]

Supplementary matherial

*Enterobacter hormaechei* BC8 16S ribosomal RNA gene sequence, SUB7603537 BC8 MT605809

3’TTAGTCTCGGGCTACGCGTTAAGTCGACCGCCTGGGGAGTACGGCCGCAAGGTTAAAACTCAAATGAATTGACGGGGGCCCGCACAAGCGGTGGAGCATGTGGTTTAATTCGATGCAACGCGAAGAACCTTACCTACTCTTGACATCCAGAGAACTTACCAGAGATGGTTTGGTGCCTTCGGGAACTCTGAGACAGGTGCTGCATGGCTGTCGTCAGCTCGTGTTGTGAAATGTTGGGTTAAGTCCCGCAACGAGCGCAACCCTTATCCTTTGTTGCCAGCGGTTAGGCCGGGAACTCAAAGGAGACTGCCAGTGATAAACTGGAGGAAGGTGGGGATGACGTCAAGTCATCATGGCCCTTACGAGTAGGGCTACACACGTGCTACAATGGCGCATACAAAGAGAAGCGACCTCGCGAGAGCAAGCGGACCTCATAAAAGTACGTCAA 5’

*Bacillus subtilis* TS2 ribosomal RNA gene sequence SUB7603302 TS2 MT605808

ACGACTTCACSCCAATCATCTGTCCCACCTTCGGCGGCTGYCTCCATAAAGGTTACCTCACCGACTTCGGGTGTTACAAACTCTCGTGGTGTGACGGGCGGTGTGTACAAGGCCCGGGAACGTATTCACCGCGGCATGCTGATCCGCGATTACTAGCGATTCCAGCTTCACGCAGTCRAGTTGCAGACTGCGATCCGAACTGAGAACAGATTTGTGGGATTGGCTTAACCTCGCGGTTTCGCTGCCCTTTGTTCTGTCCATTGTAGCACGTGTGTAGCCCAGGTCATAAGGGGCATGATGATTTGACGTCATCCCCACCTTCCTCCGGTTTGTCACCGGCAGTCACCTTAGAGTGCCCAACTGAATGCTGGCAACTAAGATCAAGGGTTGCGCTCGTTGCGGGACTTAACCCAACATCTCACGACACGAGCTGACAACAACCATGCACCACCTGTCACTCTGCCCCCRAAGGGGACGTCCTATCTCTAGGATTGTCAGARGATGTCAAGACCTGGTAARGTTCTTCGCGTTGCTTCSAATTAAACAACATGCTCCACCGCTTGTGCGGGC

*Bacillus subtilis* STL7 ribosomal RNA gene sequence,

SUB7603544 STL7 MT613864

ttaagtcccgcaacgagcgcaacccttgatcttagttgccagcattcagttgggcactctaaggtgactgccggtgacaaaccggaggaaggtggggatgacgtcaaatcatcatgccccttatgacctgggctacacacgtgctacaatggacagaacaaagggcagcgaaaccgcggttaagccaatcccacaaatctgttctcagttcggatcgcagtctgcaactcgactgcgtgaagctggaatcgctagtaatcgcggatcagcatgccgcggtgaatacgttcccgggccttgtacacaccgcccgtcacaccactagagttttgaacacccgaagtcggtgaggtaaccttttaggtgccagccgccgaaggtgggacagatgattgggg
